# Supplementary material for: Hidden heatwaves and severe coral bleaching linked to mesoscale eddies and thermocline dynamics
Source: Nat Commun. 2023 Jan 6;14:25. doi: 10.1038/s41467-022-35550-5 (PMC9822911; doi:10.1038/s41467-022-35550-5)
Supplement: Supplementary file 1 — Supplementary Information [file 41467_2022_35550_MOESM1_ESM.pdf]

Supplementary Information

for

Hidden heatwaves and severe coral bleaching linked to mesoscale eddies and thermocline dynamics

Alex S.J. Wyatt[1*], James J. Leichter[2], Libe Washburn[3,4], Li Kui[3], Peter J. Edmunds[5], Scott C. Burgess[6]

[1]Department of Ocean Science, The Hong Kong University of Science and Technology, Clear Water Bay, Kowloon, Hong Kong.

[2]Scripps Institution of Oceanography, University of California San Diego, La Jolla, California, USA.

[3]Marine Science Institute and [4]Department of Geography, University of California, Santa Barbara, California, USA.

[5]Department of Biology, California State University, Northridge, California, USA.

[6]Department of Biological Science, Florida State University, Tallahassee, Florida, USA.

[*]Correspondence to: Alex S.J. Wyatt (wyatt@ust.hk)

Surface MHW history around Moorea

Quantification of MHWs based on remotely sensed SST is sensitive to the spatial scale selected for quantification with little consistency in approach between studies. Many regional comparisons of bleaching utilise spatial scales designed to characterise SST variations across a region, such as 1 ° × 1 ° ([20]) or 2 ° × 2 ° ([31]). Conversely, some studies select small areas close to a specific site of interest, sometimes obtaining SST from a single pixel (e.g., 0.05 ° × 0.05 ° for NOAA's 'Coral Temp'). This study focused on subsurface heating on Moorea's north shore, and thus for surface comparisons we utilise a small-scale average of SST over the reef site (0.1 ° × 0.1 °, or 10 km × 10 km, consisting of an average of four pixels of 'CoralTemp' SST). However, comparisons to the broader regional pattern are interesting since it reinforces the importance of spatial scale selection and the fact that small scale variation in MHW conditions that can result from mesoscale eddy forcing.

Among the 14 regional surface MHW around the Society Islands (2 ° × 2 °) the most severe event during early 2003 lasted 23 days, corresponding to a period when local SSTs peaked at 30.6 °C and DHDs reached 15.5 °C-days regionally and 17.7 °C-days over Moorea's north shore (Fig. S1a-b; Table S3; Table S4). This led to 6-month cumulative heating of 202 °C-days in 2003. The second most severe regional surface MHW since 1985 occurred in 1994 and reached 7.0 °C-days, with 14 days above 4 °C-days and 189 cumulative °C-days. Unlike other locations, where heating and bleaching patterns tend to show a link to the phase of the El Niño Southern Oscillation (ENSO) index[78], there was no clear evidence of a strong relationship between Niño anomalies and the severity of MHW around the Society Islands (data not shown), with some strong events (2003, 2007 and 2016) occurring during a decline from strong El Niños while others were during relatively neutral periods (e.g., 1994). No significant relationships were found between the Niño 3.4 Anomaly (monthly ERSSTv5[79]) during, or in the weeks to months before, the event and the severity of the MHW as the maximum DHD.

There is some evidence that surface heating might be generally more pronounced over Moorea's north shore than regional SST would suggest. There were three MHWs not evident at a regionals scale (in 1991, 1998 and 2017) and most local events were more intense by a few °C-days or more (Fig. S1; Table S3 versus Table S4). The localisation of the 2019 MHW over Moorea's north shore was especially evident

(Fig. S1), reaching 15.4 °C-days over the north shore compared to just 2.1 °C-days regionally (Table S4 and Table S3).

SST vs. daily average in-situ temperatures

Although this study demonstrates the disconnection between surface and subsurface MHWs due to the high frequency influence of internal waves and their connection to mesoscale processes, it should be noted that at low temporal resolutions (e.g., daily), SST across scales shows evidence of being a good proxy covering the seasonal variations in temperatures experienced over the reef across depths. There were strong linear relationships between daily SST (both local and regional) and daily average in-situ temperatures, but relationships declined across depths as expected, with SST explaining between 94%, 92%, 87%, and 78% of the variation in daily average in-situ temperatures at 10, 20, 30 and 40 m, respectively. At higher resolution (e.g., 2-min data) significant temperature variability and departures from SST are evident (see Fig. 3 for in-situ temperature variability at 10, 20, 30 and 40 m).

Internal-wave cooling

It is difficult to succinctly report summary information on internal-wave cooling (IWC) experienced at Moorea due to some in-situ temperature data gaps at certain depths and times during instrument failures. Ignoring these data gaps, the maximum average IWC observed at each depth reached 0.25, 0.43, 0.56, and 0.70 °C in Dec-2006, Dec-2013, Apr-2016 and Apr-2016, respectively (Fig. S4).

Historical bleaching at Moorea

A number of bleaching events have been documented on Moorea's coral reefs prior to the recent events observed in detail in the main text here (2007, 2016 and 2019), including in 1991, 1993, 1994, 2002, and 2003[22,80-82]. Bleaching rates are difficult to directly compare among such events, especially given the marked changes in community dynamics over Moorea's reefs, because a range of factors interact to determine spatial and temporal patterns in bleaching, including changing species compositions with different susceptibility to bleaching[27,80], or apparent bleaching, including crown of thorns starfish predation. While we also lack in-situ data with which to calculate IWC during events prior to 2005, we can place them in the context of the mesoscale forcing that we have demonstrated is likely to influence IWC and thus bleaching severity. One exception is the 1991 bleaching event which occurred prior to the availability of

the SLA data product (since 1993) and during a period of limited Argo casts, but not before the daily SST product (since 1985). Despite a short 9-day surface MHW peaking at only 4.49 °C-days (Fig. S1a; Table S3), there was significant coral bleaching and some mortality reported during this event. Assessments vary, with evidence of spatial variability in bleaching and some indications that bleaching can be higher in intermediate depths[82]. Between 45 and 63 % bleaching was observed across genera[81,83], with rates as high as 76 % in *Pocillopora* spp[83]. Mortality observations ranged between 9 and 41 % across genera[81,83] and may have resulted in the highest declines in coral cover until the 2007 disturbances[84].

For subsequent bleaching events we are able to interrogate SST, SLA, and, in some cases, Argo casts to place the events in a mesoscale and potential IWC context. The 2003 MHW, which as above was the strongest surface event that has been recorded (Fig. S1a; Table S3), could be considered to have exhibited the most severe bleaching observed prior to 2019, and also occurred during a period of increasingly elevated SLA. Bleaching related mortality led to an ~10 % decline (50 % to 40 %) in coral cover across a range of water depths from 10 – 30 m[84]. Correspondingly, SLA was especially elevated around Moorea and across a broad region to the north at this time (Fig. S8d). Examined in the four pixels closest to Moorea, SLA increased rapidly leading into the peak heating period from a low in December 2002 (-5.05 cm) throughout March and April, reaching a peak of 11.9 cm on 23 April 2003. The elevated SLA during the 2003 MHW is likely to have reduced IWC, exacerbating heating and bleaching related mortality. Conversely, in 1994, a bleaching event described as being just as severe as that in 1991 led to negligible mortality[80]. This may be explained by the relatively lower SLA during much of that MHW. Observations in April 1994 during the second most severe surface MHW recorded (Table S3) suggest that 40 – 70 % of corals bleached, with bleaching evident down to 25 m[80]. *Pocillopora* sp., which dominate Moorea's contemporary reef slope, showed high rates of bleaching of between 74 and 92 % during this 1994 event[80]. Although there were sufficient casts around Moorea to provide evidence of isotherm depression, e.g., 26 °C isotherm at around 100 m depth (Table S3), that would suggest a reduction in IWC, SLAs were relatively depressed leading into the event, increasing from -5.60 cm in early Feb to a relatively mild peak of 4.83 cm on 7 April 1994. The presence of cyclonic eddies around Moorea during this MHW

(Fig. S8b) may have led to sufficient IWC to reduce subsequent coral mortality despite the high rates of bleaching.

Finally, two bleaching events that occurred outside MHW conditions provide further evidence of potentially strong links between mesoscale eddies, IWC and bleaching severity. Both the 1993 and 2002 bleaching events led to coral bleaching despite no evidence of surface MHWs in those years (Fig. S1b; Table S3). In 1993, bleaching was relatively mild[80], which may relate to relatively low SLAs at the time (Fig. S8a). SLA did increase rapidly from -0.353 cm in January 1993 to a peak of 4.35 cm on 16 Mar 1993, varying between 2.72 cm and 4.01 cm until late April, but regionally low SLA and cyclonic eddies may have reduced bleaching severity. In contrast, the 2002 bleaching event, which was also not a MHW based on SST conditions, led to about 40 – 80 % bleaching (40 – 45 % in *Pocillopora* spp.; i.e., reported as higher rates than 1991, 1994 or 2007)[22,82]. SLA was elevated around Moorea at the time with an anticyclonic eddy close to the island (Fig. S8c), leading to rapidly increasing SLA, from an albeit low level, in late December (-6.39 cm) throughout January and February to around 5-6 cm during March and April, peaking at 9.12 cm on 7 May 2002. Both the 1993 and 2002 cases demonstrate that bleaching can occur in the absence of surface MHW conditions, and that coral bleaching severity is likely to be tightly coupled to mesoscale eddy propagation and the influence of SLA on IWC.

Contemporary coral cover and bleaching observations

Coral cover data was analysed using permutational multivariate analysis of variance (PERMANOVA) within PRIMER v6 (PRIMER-E, Quest Research Limited, Aukland, NZ). PERMANOVA uses permutation of the data to construct an *F* statistic called 'pseudo-*F*'[85]. Live coral cover increased between 2016 and 2019 at both sites on the north shore fore reef (Fig. 1g-h), most significantly from a lower cover baseline at LTER 2, from 42.3 ± 1.8 % to 66.8 ± 1.7 % (pseudo-$F_{[1,77]}=102$, $p<0.001$), but also at LTER 1 where cover increased from 66.5 ± 2.2 % to 76.1 ± 1.8 % (pseudo-$F_{[1,74]}=11.4$, $p<0.005$). Including analysis of coral survey data from 2007 (Fig. 9) demonstrates that coral cover was significantly lower in 2007 than 2016 and 2019 at both LTER 1 and LTER 2 (39 ± 2.6 % and 21.5 ± 2.1 %, respectively; pseudo-$F_{[2,108]}=73.10$, $p<0.001$, and pseudo-$F_{[2,115]}=146.7$, $p<0.001$). There was a significant interaction between year and site (pseudo-$F_{[2,223]}=6.714$, $p<0.005$) so coral cover was analysed at sites separately. Conversely there was no

interaction between year and site for the percentage of corals bleached (pseudo-$F_{[1,223]}$=0.894, p=0.41) with the bleaching rate significantly higher (pseudo-$F_{[2,223]}$=158.6, p<0.0005) in 2019 (54.9 ± 1.9 %) than in 2007 (14.6 ± 2.1 %) and 2016 (14.4 ± 1.4 %).

Table S1: Comparison of heating and cooling across depths during marine heatwaves (MHWs) localised over Moorea's north shore during 2016 and 2019. Columns show the maximum continuous duration above the Maximum Monthly Mean plus 1 °C (MMM+1 = 29.8 °C, in days), maximum Degree Heating Days (DHD_{max}, in °C-days) and minimum Degree Cooling Days due to internal waves ($DCD_{IW(max)}$, in °C-days) during the respective MHWs (see Table S4 for dates). Percentage changes are shown for 2019 relative to 2016. '-' indicates an infinite change relative to 0. 'n.d.' indicates internal wave analysis not possible for sea-surface temperature (SST).

Depth (m)	Max duration > MMM+1 (days)			DHD_{max} (°C-days)			$DCD_{IW(max)}$ (°C-days)		
	2016	2019	Change	2016	2019	Change	2016	2019	Change
0 (SST)	2	11	550 %	5.73	15.38	270 %	n.d.	n.d.	n.d.
10	0.35	19.36	5600 %	3.22	16.60	520 %	-2.70	-1.48	55 %
20	0.17	5.94	3500 %	0.87	15.20	1800 %	-4.30	-1.72	40 %
30	0.04	1.40	4000 %	0.08	12.77	15000 %	-6.87	-3.02	44 %
40	0.00	0.67	-	0.00	8.26	-	-8.62	-5.48	64 %

Table S2: Summary of sea-surface temperature (SST) data coverage, the SST maximum monthly mean (MMM), and in-situ instrument deployments across depths on Moorea's north shore.

**Location (MMM)**	**Deployments**	
	**Depth (m)**	**Period**
North shore, Moorea (28.78)	0 (SST)	01 Jan 1985 – 31 Dec 2017
	10	16 Dec 2004 – 10 Aug 2019
	20	16 Dec 2004 – 10 Aug 2019
	30	16 Dec 2004 – 05 Aug 2019
	40	16 Dec 2004 – 05 Aug 2019

Table S3: Summary of the 14 regional surface marine heatwaves (MHWs) around the Society Islands since 1985. The date of the maximum degree heating day (DHD_{max}, in °C-days) calculated from satellite-derived sea-surface temperatures (SSTs) within a 2 ° × 2 ° box (see Fig. S1) is shown along with the duration in days (dates) of the event and the duration in days (dates) above 4 °C-days and 8 °C-days. Events with DHD above 4 °C-days are highlighted in bold. Shaded cells are MHW that occurred before in-situ records. '-' indicates no event.

**Event (Date)**	**DHD_{max}**	**Duration (Dates)**	**Duration > 4 °C-days (Dates)**	**Duration > 8 °C-days (Dates)**
**1 (12 Apr 1987)**	**5.03**	**33 (22 Mar 1987 – 24 Apr 1987)**	**4 (11 Apr 1987 – 15 Apr 1987)**	-
2 (23 Apr 1988)	2.30	23 (12 Apr 1988 – 05 May 1988)	-	-
3 (11 Feb 1991)	1.04	12 (11 Feb 1991 – 23 Feb 1991)	-	-
**4 (03 Mar 1991)**	**4.49**	**15 (28 Feb 1991 – 15 Mar 1991)**	**9 (03 Mar 1991 – 12 Mar 1991)**	-
**5 (04 Mar 1994)**	**7.00**	**27 (17 Feb 1994 – 16 Mar 1994)**	**14 (24 Feb 1994 – 10 Mar 1994)**	-
**6 (30 Mar 1994)**	**4.35**	**21 (21 Mar 1994 – 11 Apr 1994)**	**3 (30 Mar 1994 – 02 Apr 1994)**	-
7 (03 Apr 2001)	1.01	12 (03 Apr 2001 – 15 Apr 2001)	-	-
**8 (26 Mar 2003)**	**15.5**	**23 (15 Mar 2003 – 07 Apr 2003)**	**18 (17 Mar 2003 – 04 Apr 2003)**	**12 (20 Mar 2003 – 01 Apr 2003)**
9 (21 Feb 2007)	1.06	12 (21 Feb 2007 – 05 Mar 2007)	-	-
10 (01 Dec 2012)	1.04	12 (01 Dec 2012 – 13 Dec 2012)	-	-
11 (15 Jan 2015)	1.03	12 (15 Jan 2015 – 27 Jan 2015)	-	-
**12 (09 Apr 2016)**	**6.83**	**24 (28 Mar 2016 – 21 Apr 2016)**	**11 (07 Apr 2016 – 18 Apr 2016)**	-
13 (04 Mar 2019)	1.00	12 (04 Mar 2019 – 16 Mar 2019)	-	-
14 (05 Apr 2019)	2.10	13 (04 Apr 2019 – 17 Apr 2019)	-	-

# – Depth values are estimates extrapolated from contours with limited cast data coverage during these periods.
* – SLA values averaged over a period lacking full in-situ data coverage (5 days missing out of the 11)

Table S4: Summary of the 16 marine heatwaves (MHWs) localised over Moorea's north shore since 1985. The date of the maximum degree heating day (DHD_{max}, in °C-days) calculated from satellite-derived sea-surface temperatures (SSTs) within a 0.1 ° × 0.1 ° box (see Fig. S1) is shown along with the duration in days (dates) of the event Events with DHD above 4 °C-days are highlighted in bold. Shaded cells are MHW that occurred before in-situ records. The mean satellite and reef-level sea level anomalies (SLA in cm; see Fig. 6b), and the mean depth of the 26 °C isotherm (in m; e.g., see Fig. 7a), are shown during each event (± standard deviation). 'n.d.' indicates not data available.

DHD Event (Date)	DHD_{max}	DHD Event Duration (Dates)	Satellite SLA	Reef SLA	26 °C isotherm depth	DHW Event (Date)	DHW_{max}	DHW Event Duration (Dates)
**1 (13 Apr 1987)**	**9.34**	**34 (22 Mar 1987 – 25 Apr 1987)**	n.d.	n.d.	80.0# (±0.018)	1 (13 Apr 1987)	2.00	106 (22 Mar 1987 – 06 Jul 1987)
**2 (24 Apr 1988)**	**4.66**	**35 (12 Apr 1988 – 17 May 1988)**	n.d.	n.d.	82.5# (±0.077)	2 (05 May 1988)	0.81	107 (12 Apr 1988 – 28 Jul 1988)
3 (14 Feb 1991)	3.46	15 (11 Feb 1991 – 26 Feb 1991)	n.d.	n.d.	68.1# (±0.054)			
**4 (03 Mar 1991)**	**4.77**	**15 (28 Feb 1991 – 15 Mar 1991)**	n.d.	n.d.	67.4# (±0.133)	3 (23 Apr 1991)	1.33	155 (11 Feb 1991 – 16 Jul 1991)
5 (23 Apr 1991)	1.07	12 (23 Apr 1991 – 05 May 1991)	n.d.	n.d.	65.7# (±0.000)			
**6 (04 Mar 1994)**	**8.33**	**27 (17 Feb 1994 – 16 Mar 1994)**	-0.239 (±0.15)	n.d.	101 (±0.21)	4 (31 Mar 1994)	2.48	126 (17 Feb 1994 – 23 Jun 1994)
**7 (31 Mar 1994)**	**5.41**	**22 (21 Mar 1994 – 12 Apr 1994)**	4.03 (±0.17)	n.d.	100 (±0.093)			
**8 (25 Feb 1998)**	**4.36**	**15 (22 Feb 1998 – 09 Mar 1998)**	-7.49 (±0.12)	n.d.	59.9# (±0.009)	5 (25 Feb 1998)	0.62	87 (22 Feb 1998 – 20 May 1998)
9 (04 Apr 2001)	3.89	14 (02 Apr 2001 – 16 Apr 2001)	5.34 (±0.33)	n.d.	64.3# (±0.004)	6 (04 Apr 2001)	0.56	86 (02 Apr 2001 – 27 Jun 2001)
**10 (26 Mar 2003)**	**17.77**	**28 (14 Mar 2003 – 11 Apr 2003)**	5.80 (±0.31)	n.d.	64.9# (±0.004)	7 (30 Mar 2003)	3.02	100 (14 Mar 2003 – 22 Jun 2003)
11 (27 Feb 2007)	2.20	13 (26 Feb 2007 – 11 Mar 2007)	8.04 (±0.19)	14.2 (±0.087)	78.6# (±0.039)	8 (27 Feb 2007)	0.31	85 (26 Feb 2007 – 22 May 2007)
12 (02 Dec 2012)	3.27	14 (30 Nov 2012 – 14 Dec 2012)	1.97 (±0.31)	9.05 (±0.27)	66.4 (±0.64)	9 (02 Dec 2012)	0.47	86 (30 Nov 2012 – 24 Feb 2013)
13 (15 Jan 2015)	3.37	14 (13 Jan 2015 – 27 Jan 2015)	6.99 (±0.05)	6.22* (±0.11)	83.1 (±0.62)	10 (15 Jan 2015)	0.48	86 (13 Jan 2015 – 09 Apr 2015)
**14 (09 Apr 2016)**	**5.73**	**24 (28 Mar 2016 – 21 Apr 2016)**	9.07 (±0.15)	4.71 (±0.25)	89.1 (±1.2)	11 (09 Apr 2016)	0.82	96 (28 Mar 2016 – 02 Jul 2016)
15 (10 Jan 2017)	2.06	13 (09 Jan 2017 – 22 Jan 2017)	8.48 (±0.16)	3.16 (±0.23)	89.6 (±0.51)	12 (10 Jan 2017)	0.29	86 (09 Jan 2017 – 05 Apr 2017)
**16 (15 Apr 2019)**	**15.38**	**37 (31 Mar 2019 – 07 May 2019)**	11.3 (±0.12)	9.45 (±0.12)	130 (±0.85)	13 (25 Apr 2019)	3.16	109 (31 Mar 2019 – 18 Jul 2019)

# – Depth values are estimates extrapolated from contours with limited cast data coverage during these periods.

* – SLA values averaged over a period lacking full in-situ data coverage (5 days missing out of the 13)

Table S5: Internal-wave cooling (IWC) on Moorea's north shore during recent marine heatwaves (MHWs). For each event (see Table S3), IWC is shown in terms of the maximum (Max in °C) and average ($\overline{\mathbf{IWC}}$ in °C) of IWC during the MHW period indicated. 'n.d.' indicates no data available.

Depth (m)	MHW Event (Date Range)					
	11 (26 Feb 2007 – 11 Mar 2007)	12 (30 Nov 2012 – 14 Dec 2012)	13 (13 Jan 2015 – 27 Jan 2015)	14 (28 Mar 2016 – 21 Apr 2016)	15 (09 Jan 2017 – 22 Jan 2017)	16 (31 Mar 2019 – 07 May 2019)
Max IWC (°C)						
10	0.337	1.68	0.949	1.95	n.d.	0.473
20	0.61	1.74	n.d.	2.84	n.d.	1.11
30	1.19	1.85	n.d.	3.13	1.57	2.13
40	n.d.	2.13	2.72	2.93	1.69	3.09
$\overline{\mathrm{IWC}}$ (°C)						
10	0.0783	0.143	0.137	0.155	n.d.	0.0695
20	0.0703	0.233	n.d.	0.248	n.d.	0.0764
30	0.113	0.362	n.d.	0.440	0.216	0.150
40	n.d.	0.455	0.483	0.601	0.320	0.308

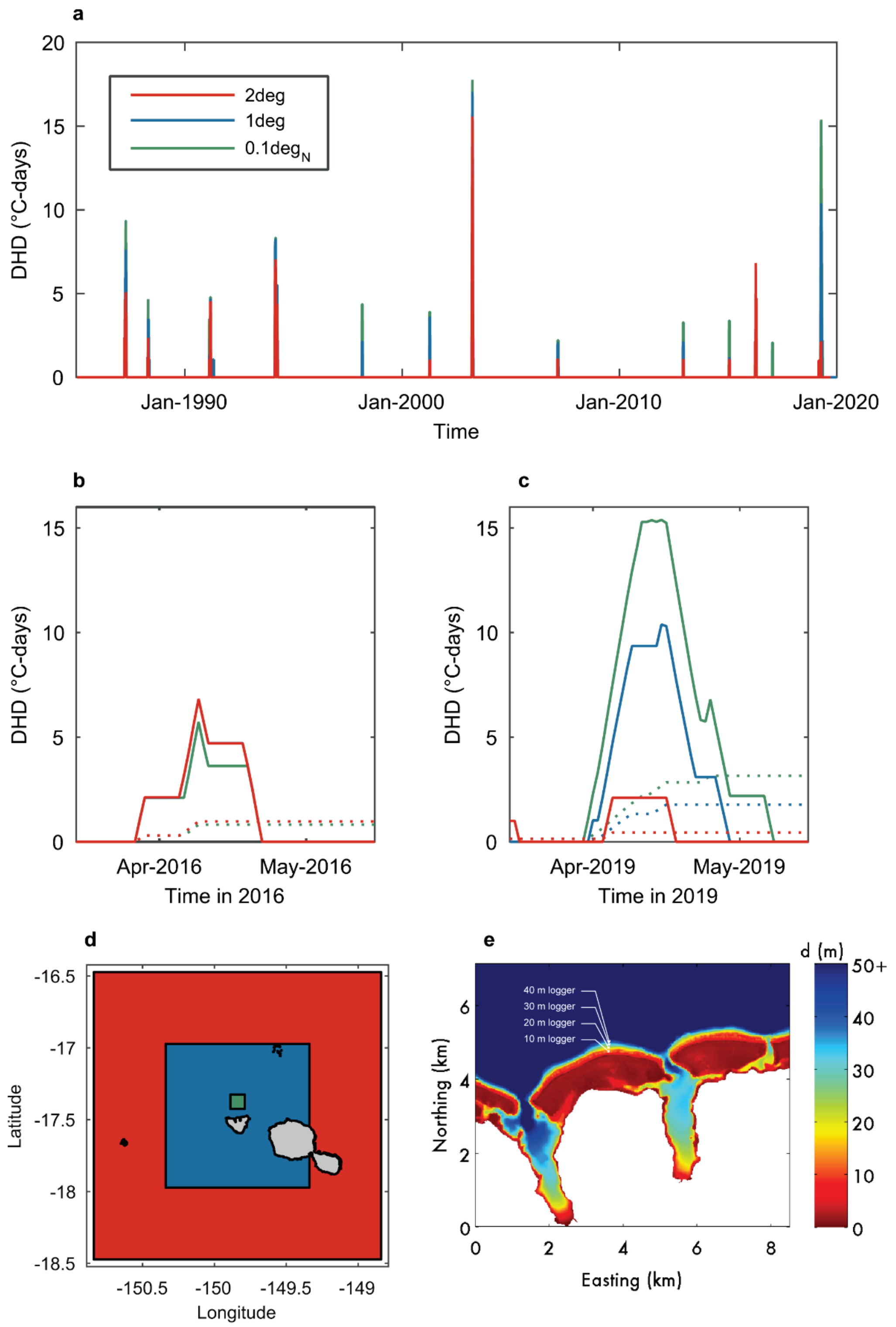

Fig. S1: Historical context for surface marine heatwaves (MHWs) around Moorea highlighting the variable effects of spatial scale selection for heating quantification. Panels show (**a**) degree heating days (DHD, °C-days) based on sea-surface temperatures (SSTs) during 1985-2019 averaged over: a (i) 2 ° × 2 ° (red; 2deg) or (ii) 1 ° × 1 ° (blue; 1deg) box around Moorea, or (iii) a 0.1 ° × 0.1 ° box over Moorea's north shore (green; 0.1deg$_N$). The spatial scale for SST averaging had (**b**) little influence on heat accumulation for the 2016 MHW, but (**c**) was markedly higher at smaller spatial scales and over a Moorea's north shore during the 2019 MHW. Dotted lines in (**b**) and (**c**) show degree heating weeks (DHW in °C-weeks) for comparison to DHD. The spatial scales can be visualised in (**d**) the map around Moorea, with (**e**) the approximate location of each in-situ loggers across depth relative to a map of reef bathymetry (depth (d) in m) from Hench[86]. Coastlines in **d** and **e** based on Wessel and Smith [77].

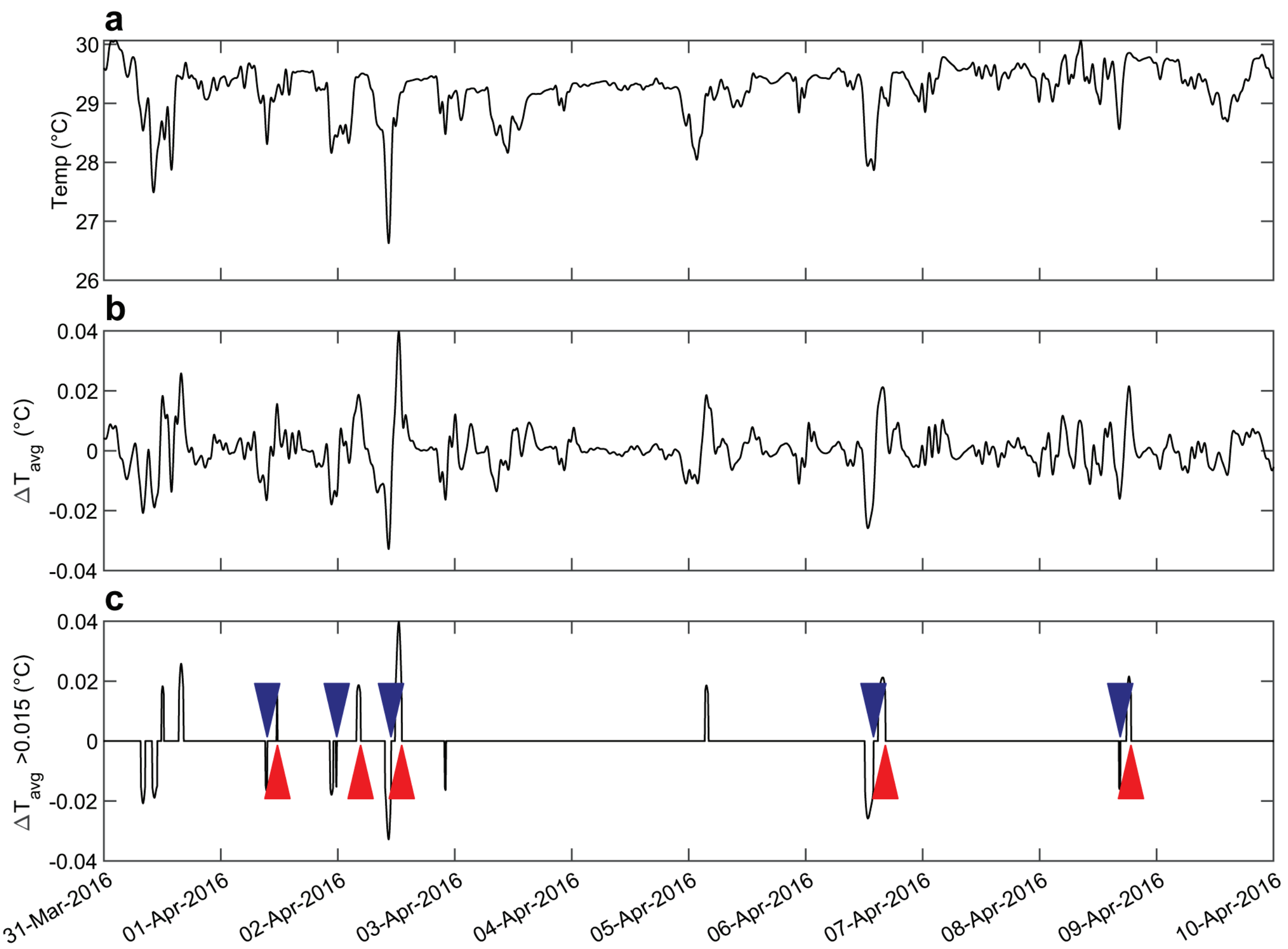

Fig. S2: Visualisation of the approach used to identify the non-linear nature of major internal wave events in in-situ temperature time series observed over the reef slope. The prevalence of cooling followed by warming back up was identified by taking (a) the water temperatures observed (black line) at reef-level (the example here is for the 20 m isobath on Moorea's north shore reef slope during 31 March to 10 April) and identifying the (b) temperature differential (averaged over a two-hour period to give ΔT_{avg}). (c) Major events were arbitrarily defined as ΔT_{avg} above or below 0.015 °C, with negative (cooling, blue) and positive (heating, red) events occurring within 10 hrs of each other identified with arrows. Cooling occurred 2.6 ± 1.3 hours before warming since 2016 (compared to 2.2 ± 0.6, 2.2 ± 1.3, at 2.0 ± 2.0 at 10, 30 and 40 m). Multiple events within close proximity (e.g., 31 Mar 2016) were excluded from the temporal analysis but showed the same trend of cooling followed by warming.

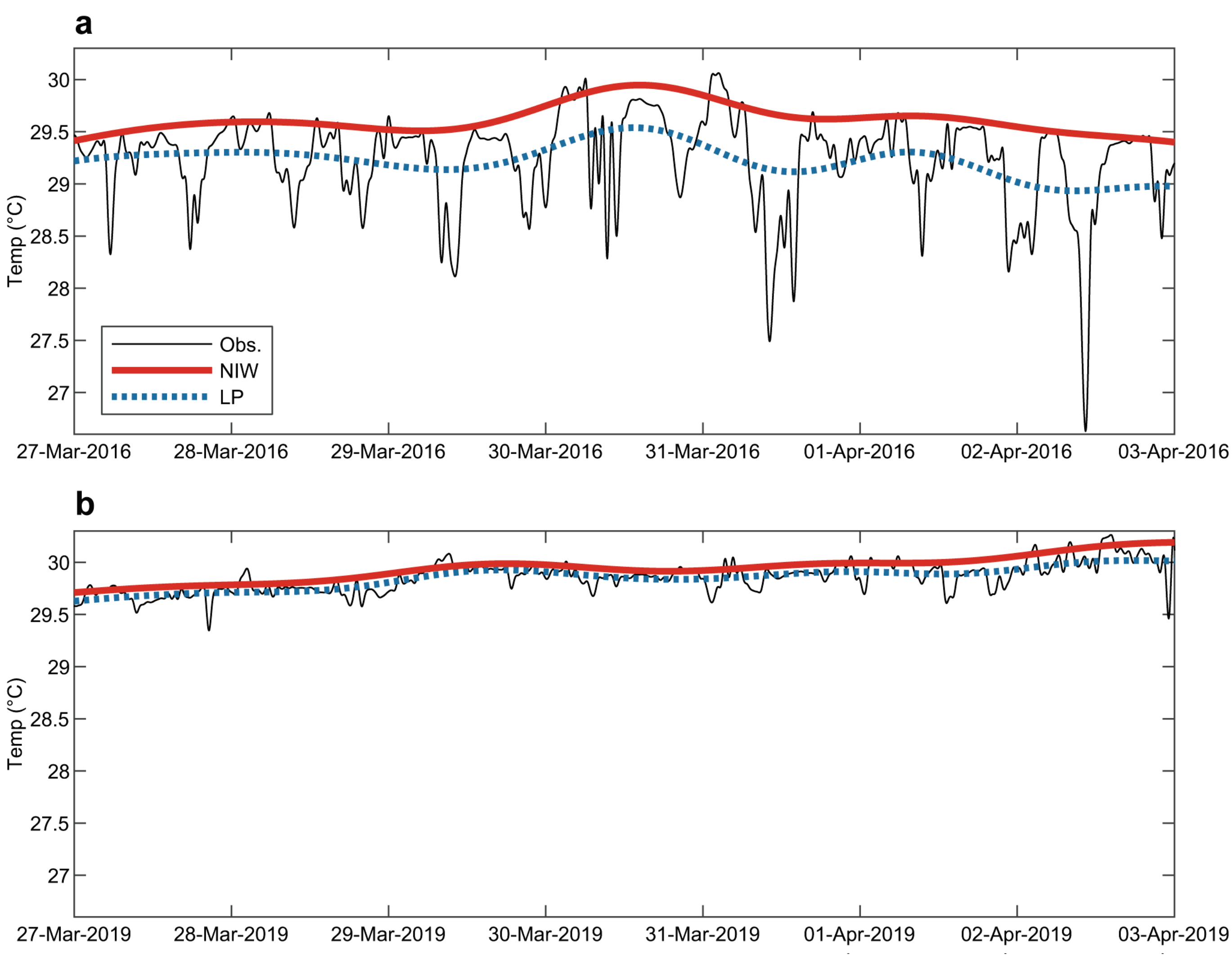

Fig. S3: Visualisation of the filtering approach described in Wyatt et al.[31] used to estimate the influence of internal waves on the in-situ temperature time series observed over the reef slope. Panels show water temperatures observed (black line) at reef-level on the 20 m isobath on Moorea's north shore reef slope during 27 March to 3 April in (**a**) 2016 and (**b**) 2019, when internal waves were prevalent and absent, respectively. The observed temperatures were low pass (LP; blue dashed line) filtered over an inertial period of 40.0 hrs at Moorea to remove variance within the semi-diurnal band. The non-internal-wave (NIW; red line) signal was then derived by shifting the LP signal upwards by an amount corresponding to the root mean square (again, within a 40.0 hr window) of the high frequency variability to reflect the observation that the influence of internal waves on temperatures observed at reef-level above the typical thermocline depth is predominately in the form of rapid, downward spikes in temperatures as the internal waves push isotherms from below the surface mixed layer up onto the reef slope (see **a**). The running mean LP signal includes the net cooling caused by internal waves and therefore tends to (**a**) underestimate prevailing temperatures in the absence of internal waves when internal waves are present in the observed signal, but (**b**) closely resembles the NIW and observed signals when internal waves are largely absent.

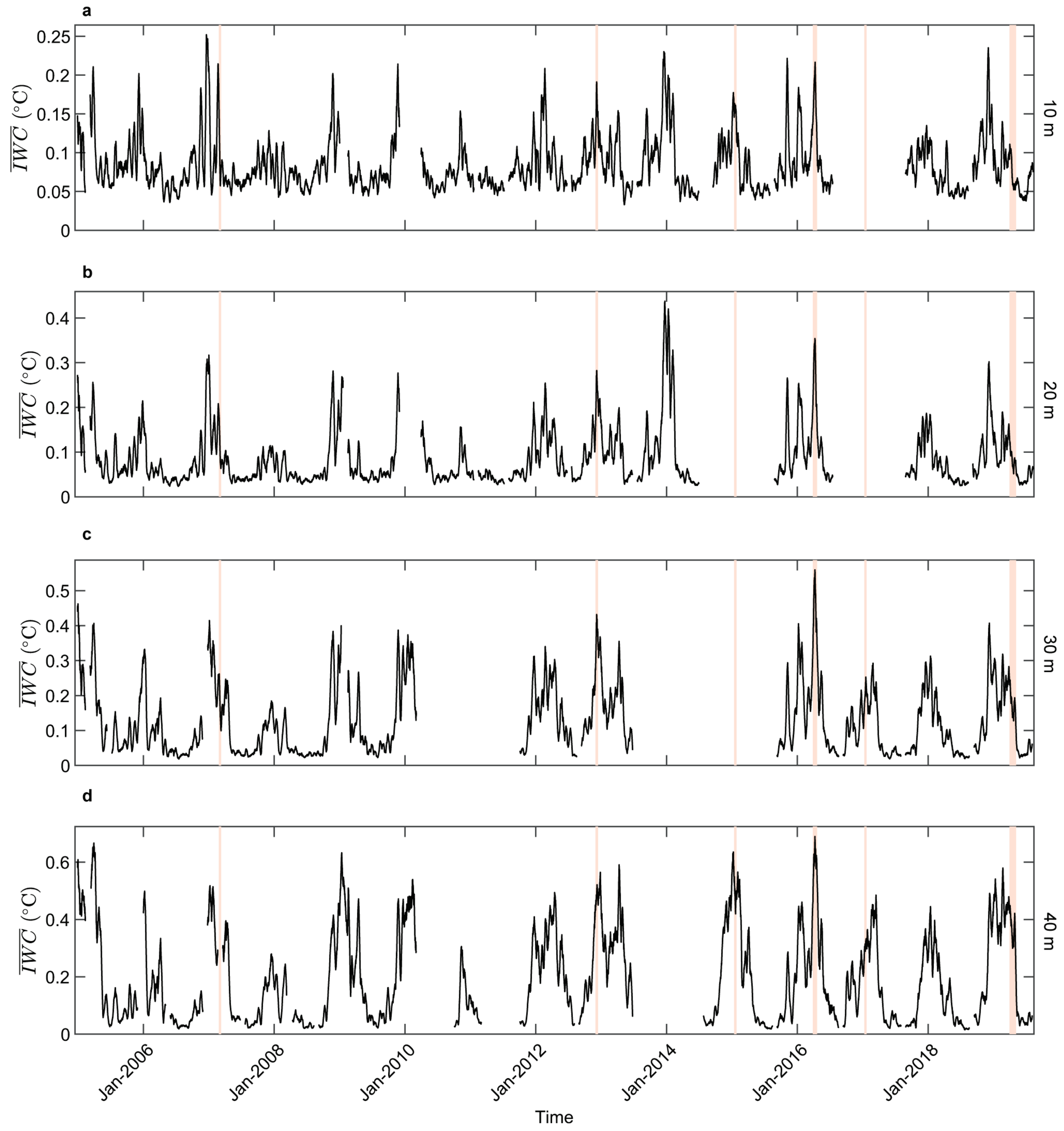

Fig. S4: Internal-wave cooling (IWC) across depths on Moorea's north shore during 2005-2019. Plots show the 12-day moving mean IWC ($\overline{\text{IWC}}$ in °C) over reefs at (**a**) 10 m, (**b**) 20 m, (**d**) 30 m and (**d**) 40 m depth**.** Local surface marine heatwave events around Moorea (see Table S4) are shown in orange.

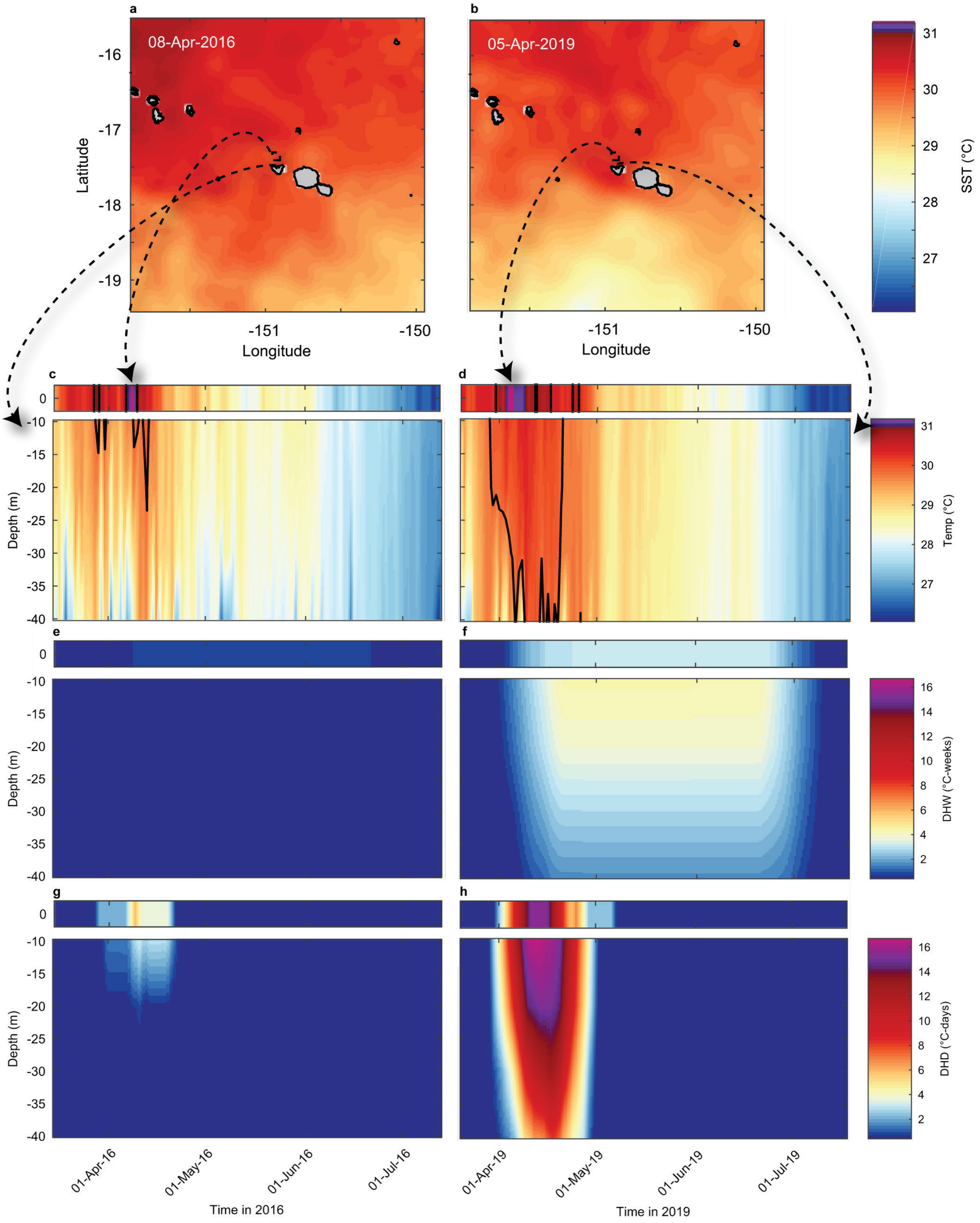

Fig. S5: Marine heatwave (MHW) severity was less pronounced and temporally offset when assessed using degree heating weeks (DHWs) instead of degree heating days (DHDs). Regional satellite sea-surface temperature (SST in °C) variability is shown over 4 × 4 ° for the peak of the Moorea MHWs in (**a**) 2016 and (**b**) 2019 (see Fig. 1 for detail and Fig. 2 for regional SST patterns). Local-scale temperature variability is shown during the (**c**) 2016 and (**d**) 2019 MHWs for SSTs averaged within 0.1 × 0.1 ° over the north shore (top panels) and in-situ temperatures measured across depths (bottom panels). The corresponding heat accumulation is shown for each MHW using (**e-f**) DHWs and, for comparison, (**g-h**) DHDs from Fig. 1, demonstrating the reduced evidence of heating and temporal offset inherent to DHWs. In-situ data in (**c-h**) were smoothed to a daily scale to match the SST resolution and for ease of visualisation. Coastlines in **a** and **b** based on Wessel and Smith[77].

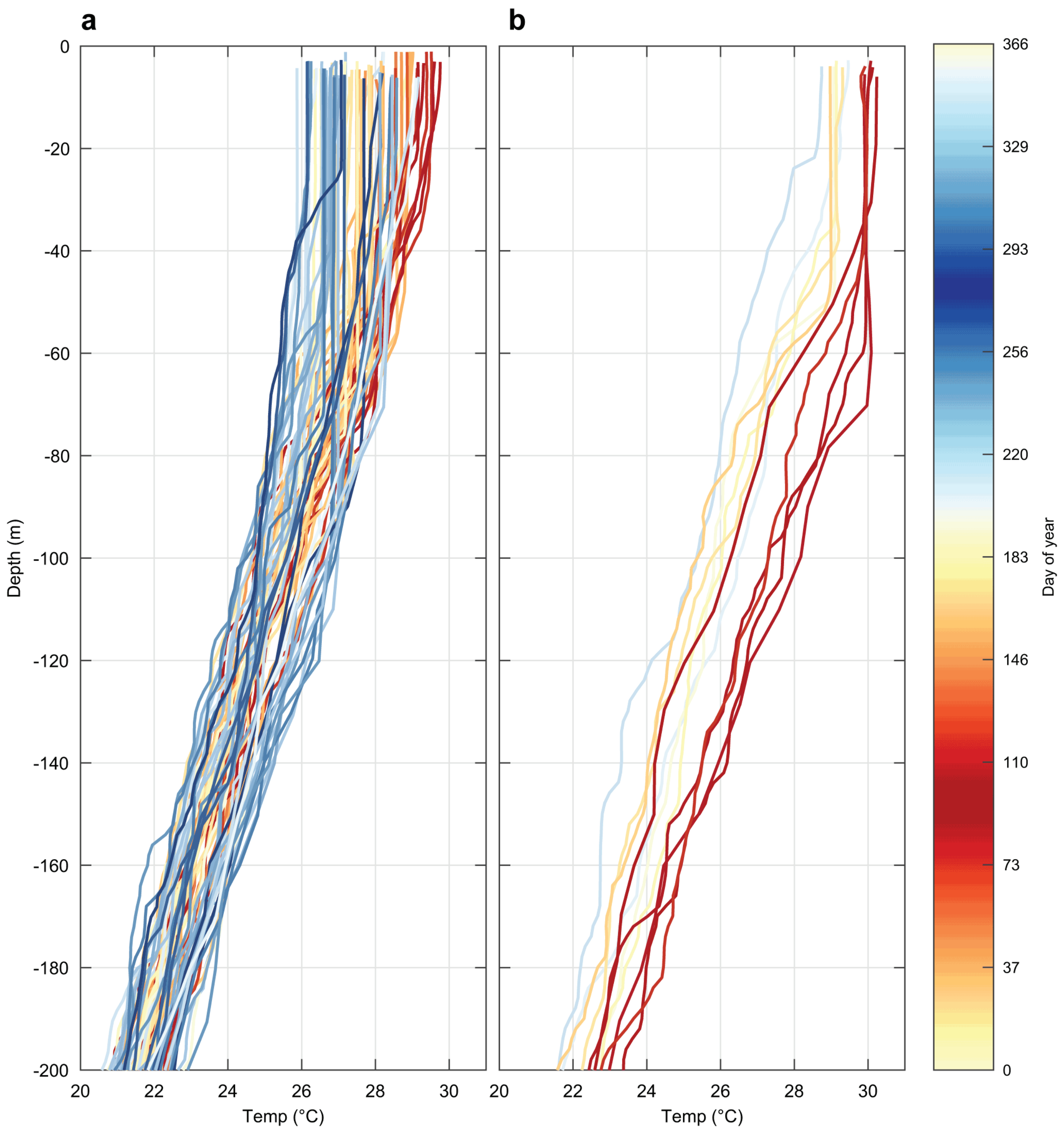

Fig. S6: Deepening of the surface layer during early 2019 based on seawater temperature profiles from Argo casts around Moorea (2 ° × 2 °) during 2016 and 2019. Profiles are shown to 200 m water depth for **a** Dec 2015 – Dec 2016 and **b** Dec 2018 – Dec 2019 with the colour scale highlighting the day of the year for each cast (e.g., Mar-Apr in red and Sep-Oct in blue).

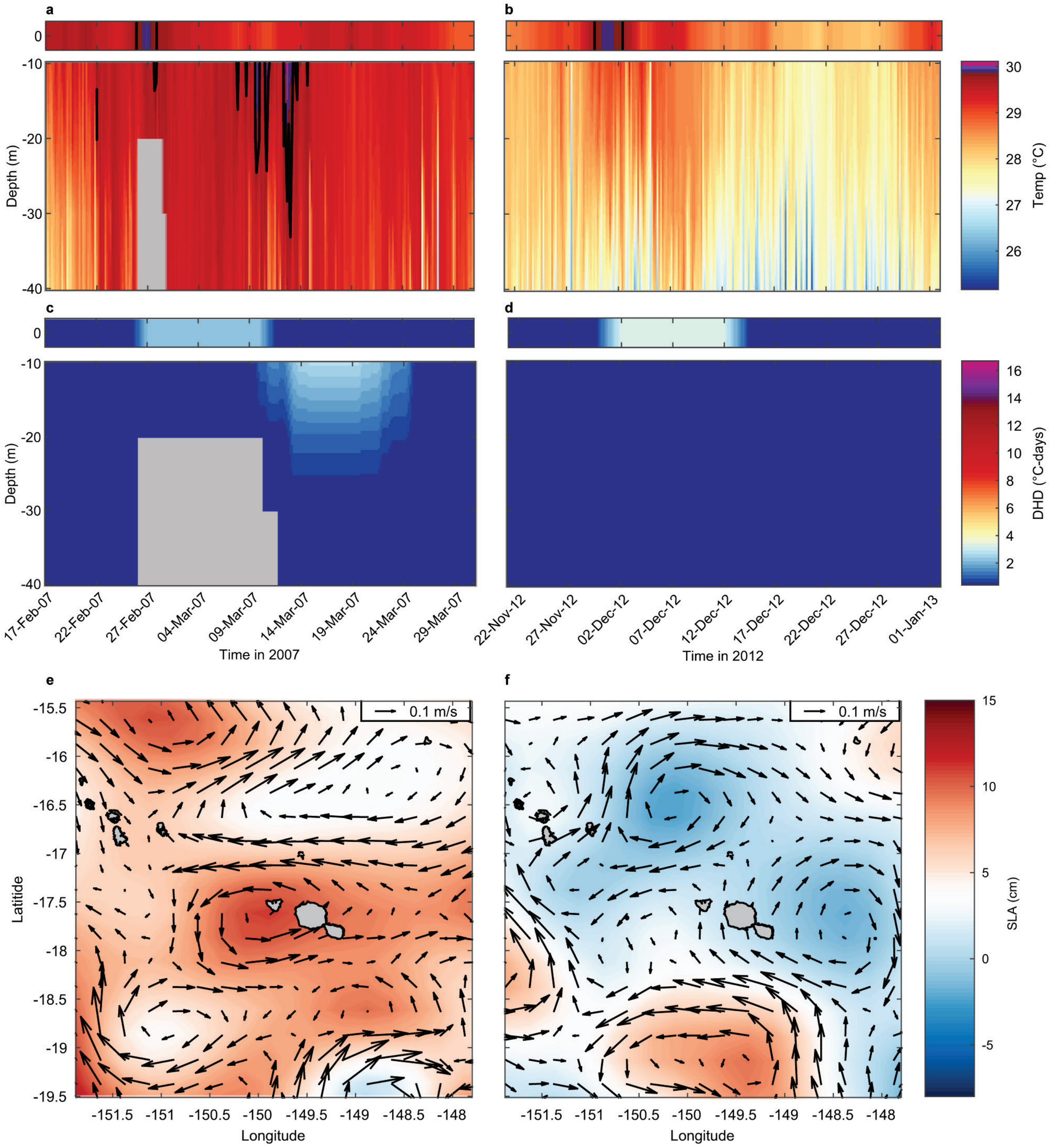

Fig. S7: Internal-wave cooling (IWC) ameliorated subsurface heating during a minor surface marine heatwave (MHW) in 2012 but not 2007 due to contrasting mesoscale eddy fields. Despite surface MHWs of similar severity, subsurface temperatures in (**a**) 2007 were higher than (**b**) 2012 due to increased IWC (see Fig. 5), leading to (**c**) a minor, shallow subsurface MHW in 2007 but (**d**) no subsurface heating in 2012. The contrasting events can be linked to mesoscale patterns in sea level anomalies (SLA in cm) shown here on (**e**) 5 March 2007 and (**f**) 10 Dec 2012 to highlight the relatively elevated and depressed sea levels, respectively, and contrasting eddy fields during the two MHWs. The black lines in **a-b** show the SST-derived bleaching threshold (29.8 °C). SST (0 m) values are shown at the top of **a-d** for comparison. Grey bar in **a** and **c** reflects a data gap. Coastlines in **e** and **f** based on Wessel and Smith[77].

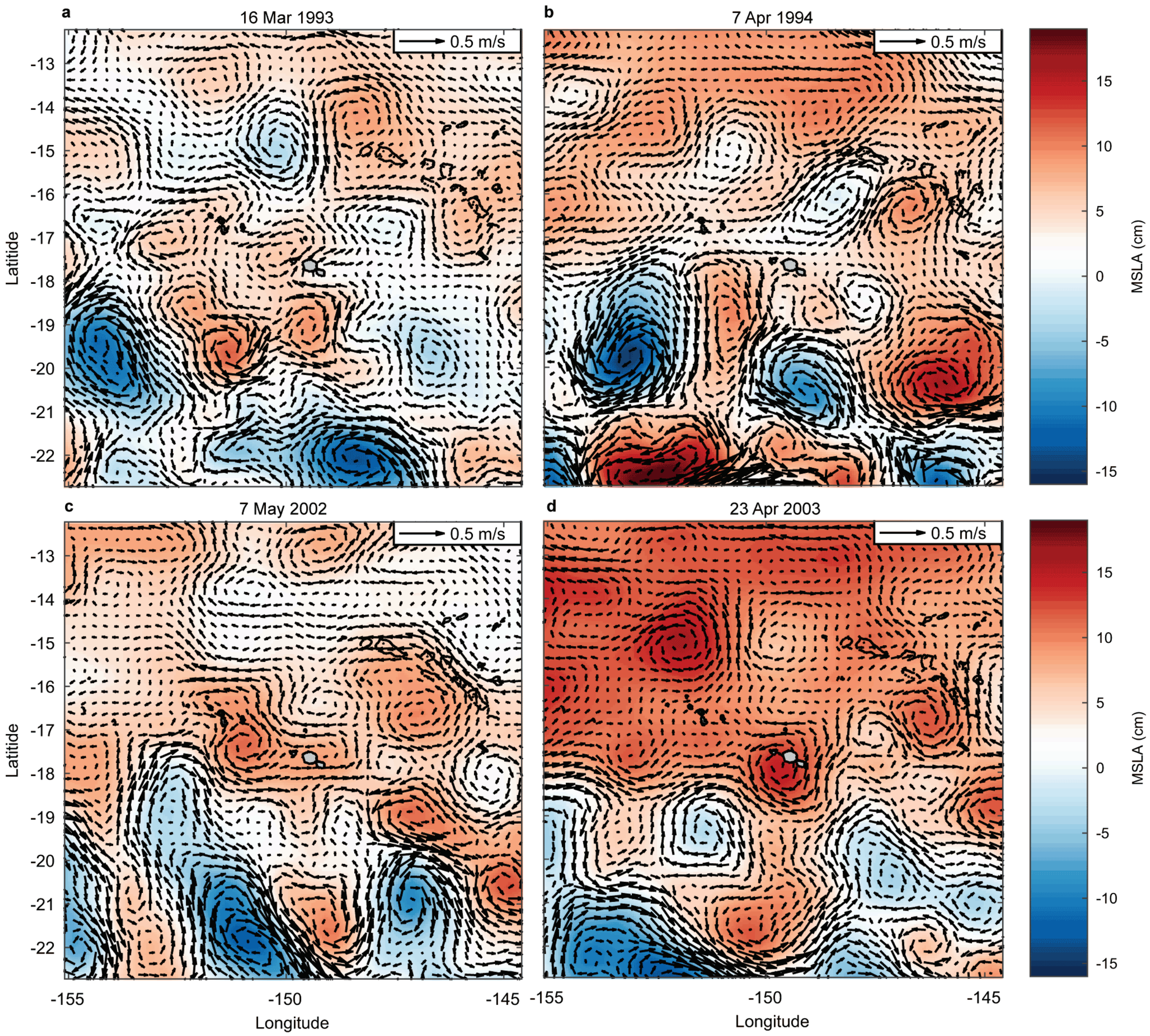

Fig. S8: Mesoscale sea-level anomalies (SLAs) around Moorea based on satellite altimetry highlighting regional differences during four historical coral bleaching events. Plots show SLAs on (**a**) 16 Mar 1993, (**b**) 7 Apr 1994, (**c**) 7 May 2002, and (**d**) 23 April 2003, highlighting (**a**) lower SLAs and cyclonic eddies around Moorea during the mild 1993 bleaching event in the absence of a surface MHW, (**b**) regional SLA elevation during the severe 1994 MHW and bleaching event, (**c**) relatively low regional SLAs but a strong anticyclonic eddy near Moorea during the 2002 bleaching event in the absence of a MHW, and (**d**) an extensive region of very high SLAs and a strong anticyclonic eddy around Moorea during the severe MHW and coral bleaching event in 2003. Dates correspond to the peak SLA around Moorea during each MHW (e.g., see Fig. 6b). Coastlines based on Wessel and Smith[77].

Supplementary References

78 Kleypas, J. A., Castruccio, F. S., Curchitser, E. N. & McLeod, E. The impact of ENSO on coral heat stress in the western equatorial Pacific. *Glob Chang Biol* **21**, 2525-2539, doi:10.1111/gcb.12881 (2015).

79 NOAA. *Monthly Atmospheric & SST Indices*, Climate Prediction Service, National Weather Service, National Oceanic and Atmospheric Administration. (2020). <https://www.cpc.ncep.noaa.gov/data/indices/> (Accessed: 14 May 2020).

80 Hoegh-Guldberg, O. & Salvat, B. Periodic mass-bleaching and elevated sea temperatures: bleaching of outer reef slope communities in Moorea, French Polynesia. *Marine Ecology Progress Series* **121**, 181-190 (1995).

81 Salvat, B. Blanchissement et mortalite des scleractiniaires sur les recits de Moorea (archipel de la Societe) en 1991. *Comptes rendus de l'Académie des sciences de Paris* **314**, 105-111 (1992).

82 Penin, L., Adjeroud, M., Schrimm, M. & Lenihan, H. S. High spatial variability in coral bleaching around Moorea (French Polynesia): patterns across locations and water depths. *Comptes Rendus Biologies* **330**, 171-181, doi:https://doi.org/10.1016/j.crvi.2006.12.003 (2007).

83 Gleason, M. G. Effects of disturbance on coral communities: bleaching in Moorea, French Polynesia. *Coral Reefs* **12**, 193-201, doi:10.1007/bf00334479 (1993).

84 Trapon, M. L., Pratchett, M. S. & Penin, L. Comparative Effects of Different Disturbances in Coral Reef Habitats in Moorea, French Polynesia. *Journal of Marine Biology* **2011**, 1-11, doi:10.1155/2011/807625 (2011).

85 Anderson, M. J. A new method for non-parametric multivariate analysis of variance. *Austral Ecology* **26**, 32-46 (2001).
