# Supplementary material for: Hidden heatwaves and severe coral bleaching linked to mesoscale eddies and thermocline dynamics
Source: Nat Commun. 2023 Jan 6;14:25. doi: 10.1038/s41467-022-35550-5 (PMC9822911; doi:10.1038/s41467-022-35550-5)
Supplement: Supplementary file 3 — Description of Additional Supplementary Files [file 41467_2022_35550_MOESM3_ESM.pdf]

Description of Additional Supplementary Files:

Supplementary Movie 1: Animation of sea-surface temperatures (SSTs) in a 4 ° x 4 ° box surrounding Moorea, French Polynesia from 15 March to 15 May in 2016 and 2019

Supplementary Movie 2: Animation of sea-surface temperatures (SSTs) in a 10 ° x 10 ° box surrounding Moorea, French Polynesia from 15 March to 15 May in 2016 and 2019

Supplementary Movie 3: Animation of sea level anomalies (SLAs) in a 4 ° x 4 ° box surrounding Moorea, French Polynesia from 15 March to 15 May in 2016 and 2019

Supplementary Movie 4: Animation of sea level anomalies (SLAs) in a 10 ° x 10 ° box surrounding Moorea, French Polynesia from 15 March to 15 May in 2016 and 2019
